# Socioeconomic differences in contact reduction in the first COVID-19-wave in Germany (CoMoLo-study)

**DOI:** 10.1093/eurpub/ckac131.194

**Published:** 2022-10-25

**Authors:** F Beese, B Wachtler, S Haller, C Koschollek, T-K Pfoertner, J Hoebel

**Affiliations:** 1 Department of Epidemiology and Health Monitoring, Robert Koch Institute, Berlin, Germany; 2 Department of Infectious Disease Epidemiology, Robert Koch Institute, Berlin, Germany; 3 IMVR, University of Cologne, Cologne, Germany

## Abstract

**Background:**

The COVID-19 pandemic has led to physical distancing measures across a range of countries to control the spread of the virus. Evidence referring to contact dynamics in different socioeconomic populations is still sparse and may contribute to the explaining of socioeconomic inequality of infections.

**Methods:**

Data came from two early COVID-19 hotspots in Germany using the CORONA-MONITORING-lokal study (CoMoLo). The sample (n = 3,637) was restricted to working age (18-67 years). We calculated the association of individual education and occupation status (low, middle, high) and self-reported private and professional contact reductions. Using weighting factors (adaptation to local age, gender and education distribution), we performed multivariate Poisson regressions (prevalence ratios; PR) with different sets of covariates: hotspot, age, sex, country of birth, household size, contact level before physical distancing measures and home office.

**Results:**

The descriptive analyses show a clear socioeconomic gradient in private (low education: 70,0%; middle: 79,1%; high: 86,2%) and professional contact reductions (low education: 54,6%; middle: 61,3; high: 77,2%). The multivariate analyses confirm these associations, with a stronger gradient for professional contacts (private contact reduction: PR low vs. high education = 0,83 [KI:0.74-0.93]; professional contact reduction: PR low vs. high education = 0,75 [KI:0.64-0.89]) as well as for professional contact reduction when occupational status is considered instead of education.

**Conclusions:**

Our results show disadvantages in groups with lower educational or occupational status in private and professional contact reductions in the first pandemic wave. This might result in a higher risk of infection. Preventive measures that a) adequately explain the importance of contact restrictions and b) facilitate the implementation of these reductions seem necessary to better protect structurally disadvantaged people during epidemics.

**Key messages:**

• Groups with lower educational or occupational status were less likely of being able to reduce their private and professional contacts in the first wave of the pandemic.

• Socioeconomic differences were more pronounced in professional contact reduction compared to private contact reduction.

